# High prevalence of hip lesions secondary to arthroscopic over- or undercorrection of femoroacetabular impingement in patients with postoperative pain

**DOI:** 10.1007/s00330-021-08398-4

**Published:** 2021-11-29

**Authors:** Malin K. Meier, Till D. Lerch, Simon D. Steppacher, Klaus A. Siebenrock, Moritz Tannast, Peter Vavron, Ehrenfried Schmaranzer, Florian Schmaranzer

**Affiliations:** 1 Department of Orthopedic Surgery and Traumotology, Inselspital Bern University Hospital, University of Bern, Freiburgstrasse, 3010 Bern, Switzerland; 2 Department of Diagnostic-, Interventional- and Pediatric Radiology, Inselspital Bern University Hospital, University of Bern, Freiburgstrasse, 3010 Bern, Switzerland; 3 Department of Orthopaedic Surgery, Fribourg Cantonal Hospital, University of Fribourg, Chemin des Pensionnats 2-6, 1752 Villars-sur-Glâne, Switzerland; 4 Department of Orthopaedic Surgery, District Hospital St. Johann in Tirol, Bahnhofstrasse 14, 6380 St. Johann in Tirol, Austria; 5 Department of Radiology, District Hospital St. Johann in Tirol, Bahnhofstrasse 14, 6380 St. Johann in Tirol, Austria

**Keywords:** MR arthrography, Hip arthroscopy, Cartilage, Hip, Femoroacetabular impingement

## Abstract

**Objectives:**

To compare the prevalence of pre- and postoperative osseous deformities and intra-articular lesions in patients with persistent pain following arthroscopic femoroacetabular impingement (FAI) correction and to identify imaging findings associated with progressive cartilage damage.

**Methods:**

Retrospective study evaluating patients with hip pain following arthroscopic FAI correction between 2010 and 2018. Pre- and postoperative imaging studies were analyzed independently by two blinded readers for osseous deformities (cam-deformity, hip dysplasia, acetabular overcoverage, femoral torsion) and intra-articular lesions (chondro-labral damage, capsular lesions). Prevalence of osseous deformities and intra-articular lesions was compared with paired *t*-tests/McNemar tests for continuous/dichotomous data. Association between imaging findings and progressive cartilage damage was assessed with logistic regression.

**Results:**

Forty-six patients (mean age 29 ± 10 years; 30 female) were included. Postoperatively, 74% (34/46) of patients had any osseous deformity including 48% (22/46) acetabular and femoral deformities. Ninety-six percent (44/46) had an intra-articular lesion ranging from 20% (9/46) for femoral to 65% (30/46) for acetabular cartilage lesions. Prevalence of hip dysplasia increased (2 to 20%, *p* = 0.01) from pre- to postoperatively while prevalence of cam-deformity decreased (83 to 28%, *p* < 0.001).

Progressive cartilage damage was detected in 37% (17/46) of patients and was associated with extensive preoperative cartilage damage > 2 h, i.e., > 60° (OR 7.72; *p* = 0.02) and an incremental increase in postoperative alpha angles (OR 1.18; *p* = 0.04).

**Conclusion:**

Prevalence of osseous deformities secondary to over- or undercorrrection was high. Extensive preoperative cartilage damage and higher postoperative alpha angles increase the risk for progressive degeneration.

**Key Points:**

*• The majority of patients presented with osseous deformities of the acetabulum or femur (74%) and with intra-articular lesions (96%) on postoperative imaging.*

*• Prevalence of hip dysplasia increased (2 to 20%, p = 0.01) from pre- to postoperatively while prevalence of a cam deformity decreased (83 to 28%, p < 0.001).*

*• Progressive cartilage damage was present in 37% of patients and was associated with extensive preoperative cartilage damage > 2 h (OR 7.72; p = 0.02) and with an incremental increase in postoperative alpha angles (OR 1.18; p = 0.04).*

**Supplementary Information:**

The online version contains supplementary material available at 10.1007/s00330-021-08398-4.

## Introduction

Recent years have led to an exponential increase in the number of hip arthroscopies performed for treatment of osseous deformities and intra-articular lesions secondary to femoroacetabular impingement [1]. This development has been fostered by innovations in surgical techniques, improved preoperative imaging, and the widely reported short- to long-term benefit of FAI surgery [2–4]. Despite the favorbale surgical outcome of FAI correction up to 18% of patients present with postoperative pain and up to 10% reportedly undergo revision surgery within 2 years [5, 6]. These patients are exposed to a substantially higher risk for worse surgical outcome following revision surgery than patients who do not require further surgical treatment [7]. Further cross-sectional imaging is commonly recommended to diagnose deformities resulting from surgical over- or undercorrection such as residual cam deformities and intra-articular lesions [8, 9]. However, imaging assessment is challenging in the setting of postoperative hip pain due to the difficulties in identifying the relevant osseous deformities and differentiating normal postoperative findings from relevant pathology [8, 10, 11].

Currently, few studies have assessed the prevalence of osseous deformities following arthroscopic FAI surgery with cross-sectional imaging [12]. MR arthrography has been used in the setting of postoperative hip pain to detect recurrent labrum lesions, cartilage damage, and capsular lesions [10, 13–15]. However, to date, the prevalence of osseous deformities and intra-articular lesions including their potential association following failed hip arthroscopy is unclear which would provide useful information for treatment planning.

Thus, the aim of this study was to compare the prevalence of osseous deformities and intra-articular lesions in patients with persistent pain following arthroscopic FAI correction between pre- and postoperative imaging and to identify imaging findings associated with progressive cartilage damage and postoperative labrum lesions.

## Material and methods

### Patients

Following IRB approval and a waiver for written informed consent, a retrospective study was performed at a primary orthopedic hospital. The imaging database was searched for consecutive patients originating from Western Austria who had undergone arthroscopic FAI correction and preoperative radiographic and traction MR arthrography according to the institutional routine protocol between January 2010 and 2018. Inclusion criterion was availability of postoperative imaging including radiographs and traction MR arthrography for refractory hip pain. Patients were excluded if no sequences covering the distal femoral condyles for measurement of femoral torsion were available. Diagnosis of hip pain was established by two arthroscopic hip surgeons based on a > 3-month history of symptoms and a positive impingement test [2, 14].

### Diagnostic imaging

AP pelvis and Dunn 45° views were obtained in supine position pre- and postoperatively [16]. Pre- and postoperative MR arthrography was performed at 1.5 T (Magnetom Symphony/Aera; Siemens Healthineers) following fluoroscopic injection of 1–2 ml of iodinated contrast agent (iopamidol, 200 mg/ml; Iopamiro 200; Bracco), 2–5 ml of local anesthetic (ropivacaine hydrochloride; 2 mg/ml; Ropinaest;Gebro Pharma), and 15–20 ml of diluted MR contrast agent (gadopentetate dimeglumine, 2 mmol/l; Magnevist; Bayer Healthcare). As part of the institutional routine protocol, leg traction was applied during MRI using a previously described method and a dedicated traction device (TRACView; Menges Medical) [17, 18]. This includes a supporting plate for stabilization of the contralateral leg, a weight (adjusted to patients constitution: 15 kg for patients < 60 kg, 18 kg for patients 60–80 kg, 23 kg for patients > 80 kg) connected to a cable whinch via a pulley which is connected to an ankle brace. The preoperative imaging protocol included multiplanar (coronal, sagittal, and axial-oblique) gradient echo- or turbo spin echo sequences and 3D sequences for reformation of radial images with a total imaging time of 21–23 min. The postoperative imaging protocol included multiplanar turbo spin echo sequences and 3D sequences for reformation of radial images. In addition, axial sequences of the pelvis and distal femoral condyles were acquired without leg traction for measurement of femoral torsion leading to a total imaging time of 23–25 min. Imaging protocol is given in Supplementary Table 1.

### Image analysis

Analysis of pre- and postoperative imaging was performed independently by two blinded readers (radiologist with 12 years (E.S.) and resident with 7 years (F.S.) of experience in hip imaging). Pre-and postoperative imaging studies were compared directly against each other, blinded to the operative records. Radiographs were assessed for Tönnis grade of osteoarthritis, lateral center edge (LCE) angle according to Wiberg et al. [19], acetabular index, and acetabular retroversion signs (cross over, posterior wall, ischial spine signs) [16, 20]. Diagnosis of osseous deformities was made according to the 2020 Lisbon agreement on FAI imaging [20]: hip dysplasia = LCE < 25°, mild acetabular overcoverage LCE 34–40°, severe acetabular overcoverage = LCE > 40°, acetabular retroversion = presence of all 3 retroversion signs. On radial images, maximum alpha angles were measured and angles > 60° were consistent with a cam deformity [20]. Femoral torsion was measured according to the method described by Murphy et al. [21] and angles < 0° and > 35° were used to diagnose femoral retrotorsion and excessively high femoral torsion, respectively [22].

Acetabular and femoral cartilage damage was consistent with delamination, thinning, or defect [23]. Presence of extensive cartilage damage > 2 h on the clock face (i.e., > 60°) was recorded as it has been linked with failure of FAI surgery [20, 24]. Pre- and postoperative imaging was compared to assesses progressive cartilage damage which was defined as any new acetabular/femoral cartilage lesion on postoperative imaging and extension of cartilage damage > 2 h in a patient with cartilage damage < 2 h on preoperative imaging. Diagnosis of a postoperative labrum lesion was made as described previously [15]: contrast extension to the labrum surface, presence of paralabral cyst or extension of labrum abnormality to a new location on postoperative MRI. Presence or absence of capsular defects and adhesions was assessed [11]. Obliteration of the paralabral sulcus was not recorded as it reportedly is a uniform postoperative finding [11].

### Statistical analysis

Normal distribution of continuous data was confirmed using Kolmogorov Smirnov test.

Prevalence of osseous deformities and intra-articular lesions was compared pre- and postoperatively with paired *t*-tests for continuous and McNemar tests for dichotomous data, respectively. Post hoc sample size calculation was performed for assessment of interrater reliability using Cohen’s kappa (ĸ). Fair agreement consistent with a ĸ > 0.2 was chosen as minimum level of agreement. Substantial agreement corresponding to a ĸ of 0.7 was defined as expected interrater agreement [25]. Assuming a significance level of 0.05, a power of 0.80 and an expected proportion ranging from 0.2 to 0.8 to account for the multiple outcome parameters led to a minmum sample size of 40 hips [26]. After confirmation of at least moderate (κ > 0.4) interrater reliability for all parameters, results of reader 1 were used for logistic regression analysis [25]. Association of progressive cartilage damage and postoperative labrum lesions with demographic factors and imaging findings was evaluated with odds ratios and corresponding 95% confidence intervals (CIs) using logistic regression analyis. If more than two significant associations were found, a multivariate logistic regression analysis was performed. A type I error rate of 5% was used to determine statistical significance. Statistical analysis was performed using GraphPad Prism (Version 9.1, GraphPad Software).

## Results

### Patient characteristics

Of the 806 patients with preoperative imaging and subsequent hip arthroscopy at our institution, 72 patients met the inclusion criteria. Twenty-six patients were excluded due to lack of images of the distal femoral condyles. Finally, 46 patients (46 hips, 30 female) with a mean age at surgery of 29 ± 10 years (age range, 16–54 years) and complete pre- and postoperative imaging were included who underwent different arthroscopic procedures (Table 1). Mean time between surgery and postoperative MRI was 1.4 ± 1.0 years (range 0.1–4.9 years). Seventeen percent (8/46) had revision surgery 1.6 ± 0.8 years after the index procedure and 7% (3/46) underwent subsequent total hip replacement within 2 years (Table 1).

**Table 1:** Demographic characteristics

Characteristic	Study group 46 patients (46 hips)
Age at surgery (year)	29 ± 10
Time between surgery and second MRI (year)	1.4 ± 1
Female sex	65% (30/46)
Surgical procedure	
Cam resection	93% (43/46)
Labrum debridement	11% (5/46)
Labrum refixation	76% (35/46)
Labrum reconstruction	9% (4/46)
Acetabular rim trimming	72% (33/46)
Subspine decompression	2% (1/46)
Cartilage repair	5% (2/46)
Subsequent revision surgery	17% (8/46)
Cam resection	38% (3/8)
Labrum debridement	50% (4/8)
Labrum refixation	50% (4/8)
Labrum reconstruction	13% (1/8)
Acetabular rim trimming	50% (4/8)
Adhesioloysis and caspsular closure	88% (7/8)
Derotational femoral osteotomy	13% (1/8)
Subsequent total hip arthroplasty	7% (3/46)

### Prevalence of osseous deformities

Postoperatively, 74% (reader 1, 34/46) and 65% (reader 2, 30/46) of patients had an osseous deformity of the acetabulum or the femur. An acetabular deformity was found in 48% (22/46) of patients for reader 1 and 41% (19/46) of patients for reader 2 (Table 2). A femoral deformity was found in 48% (22/46) of patients for reader 1 and 41% (19/46) of patients for reader 2 (Table 2).

**Table 2:** Comparison of prevalence between preoperative and postoperative osseous deformities and intra-articular lesions

Parameter	Reader 1				Reader 2			
	Preoperative	Postoperative	Difference	*p* value	Preoperative	Postoperative	Difference	*p* value
Osseous deformities								
Tönnis grade = 1	30% (14)	41% (19)	11% (2–20%)	0.06	21% (10)	33% (15)	12% (3–21%)	0.06
LCE (°)	34 ± 6	31 ± 7	3 ± 4 (2–5)	< 0.001	32 ± 6	29 ± 7	3 ± 4 (2– 4)	< 0.001
Hip dysplasia (LCE < 25°)	2% (1)	20% (9)	18% (7–29%)	0.01	4% (2)	22% (10)	18% (7–29%)	0.01
Mild overcoverage (LCE 34–40°)	22% (10)	15% (7)	7% (0–14%)	0.51	17% (8)	13% (6)	4% (− 2–10%)	0.69
Severe overcoverage (LCE > 40°)	20% (9)	13% (6)	7% (0–14%)	0.25	15% (7)	9% (4)	6% (0–13%)	0.25
Acetabular index (°)	4 ± 5	6 ± 5	2 ± 6 (0–4)	0.04	5 ± 4	6 ± 5	1° ± 4° (0–2)	0.03
Acetabular retroversion	13% (6)	2% (1)	11% (2–20%)	0.06	15% (7)	4% (2)	11% (2–20%)	0.06
Crossover sign	65% (30)	52% (24)	13% (3–23%)	0.03	63% (29)	41% (19)	22% (10–34%)	0.01
Posterior wall sign	57% (26)	52% (24)	5% (0–11%)	0.63	41% (19)	28% (13)	13% (3–23%)	0.03
Ischial spine sign	33% (15)	33% (15)	0	1.00	28% (13)	22% (10)	6% (0–13%)	0.38
Alpha angle (°)	71 ± 9	60 ± 11	11 ± 11 (7–14)	< 0.001	70 ± 11	60 ± 10	10 ± 11 (7–14)	< 0.001
Cam deformity	83% (38)	28% (13)	55% (41–69%)	< 0.001	76% (35)	22% (10)	54% (40–68%)	< 0.001
Femoral torsion (°)		24 ± 11				26 ± 12		
Increased femoral torsion (> 35°)		24% (11)				20% (9)		
Decreased femoral torsion (< 0°)		4% (2)				4% (2)		
Intra-articular lesions								
Labrum lesion	93% (43)	48% (22)	45% (31–59%)	< 0.001	98% (45)	50% (23)	48% (34–62%)	< 0.001
Acetabular cartilage lesion	54% (25)	65% (30)	11% (2–20%)	0.06	70% (32)	80% (37)	10% (1–19%)	0.06
Cartilage lesions > 2 h	24% (11)	37% (17)	13% (3–23%)	0.07	20% (9)	41% (19)	21% (9–33%)	0.01
Femoral cartilage lesion	7% (3)	20% (9)	13% (3–23%)	0.03	9% (4)	17% (8)	9% (1–17%)	0.13
Progressive cartilage damage	–	37% (17)	–	–	–	37% (17)	–	–
Capsular defect	–	48% (22)	–	–	–	59% (27)	–	–
Capsular adhesion	–	39% (18)	–	–	–	48% (22)	–	–

*LCE* lateral center edge angle

**Fig. 1:**
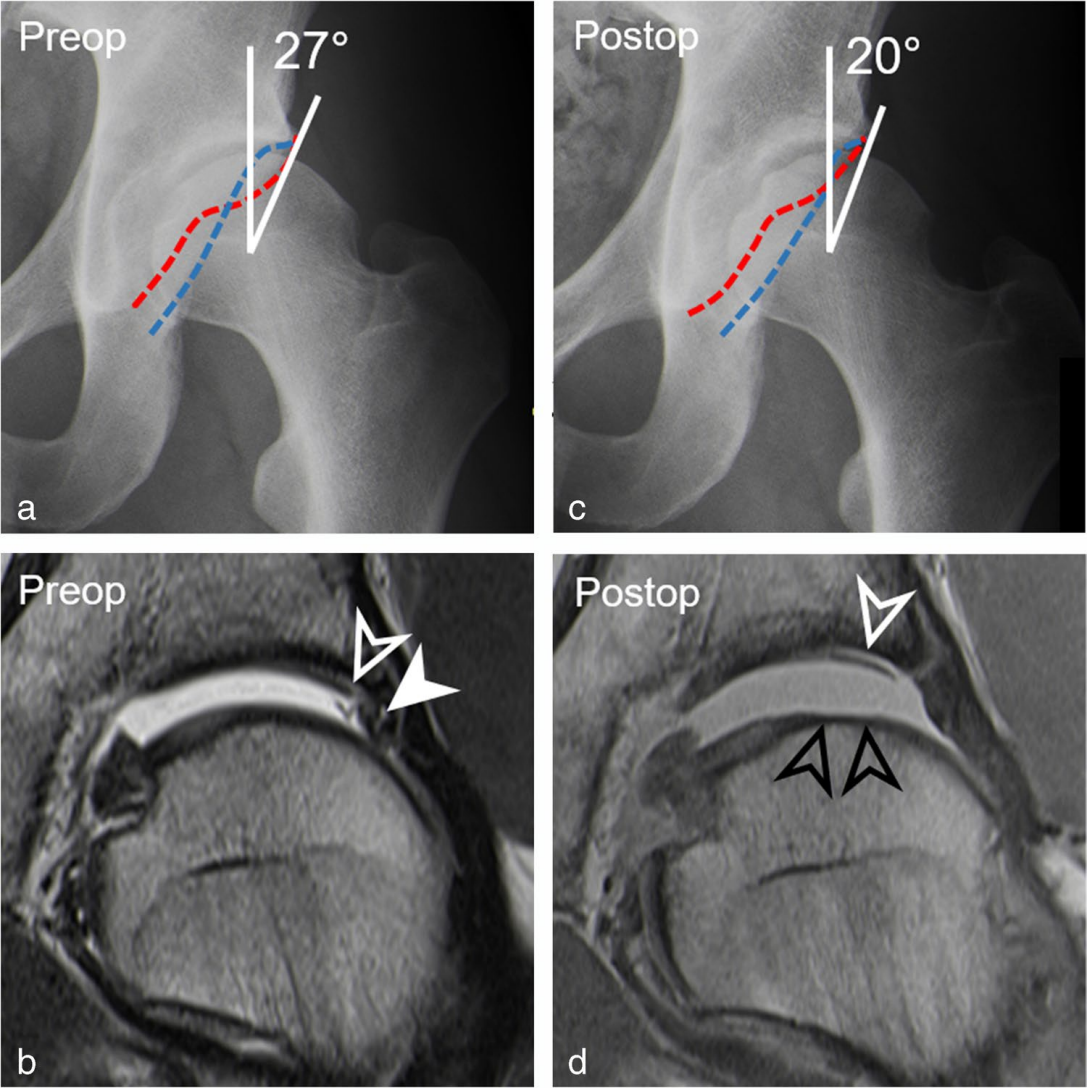
A 23-year-old man with persisting pain 14 months after arthroscopic cam resection and acetabular rim trimming with labrum refixation. **a** Preoperative AP pelvis view shows normal lateral coverage (LCE: 27°), a cross-over sign, and a cam deformity. **b** Preoperative coronal T1-w TSE image (repetition time/echo time, 450 ms/12 ms) shows labrum lesion (filled arrow head) and cartilage delamination (arrowhead). **c** Postoperative AP pelvis view shows a dysplastic actabulum (LCE: 20°) following overcorrection. **d** Postoperative coronal PD-w TSE image (2460 ms/ 13 ms) shows large cartilage flap (white arrowhead) and new femoral cartilage defect (black arrowheads) indicating progressive cartilage damage. Note: anterior acetabular wall (red dotted line), posterior acetabular wall (blue dotted line)

**Fig. 2:**
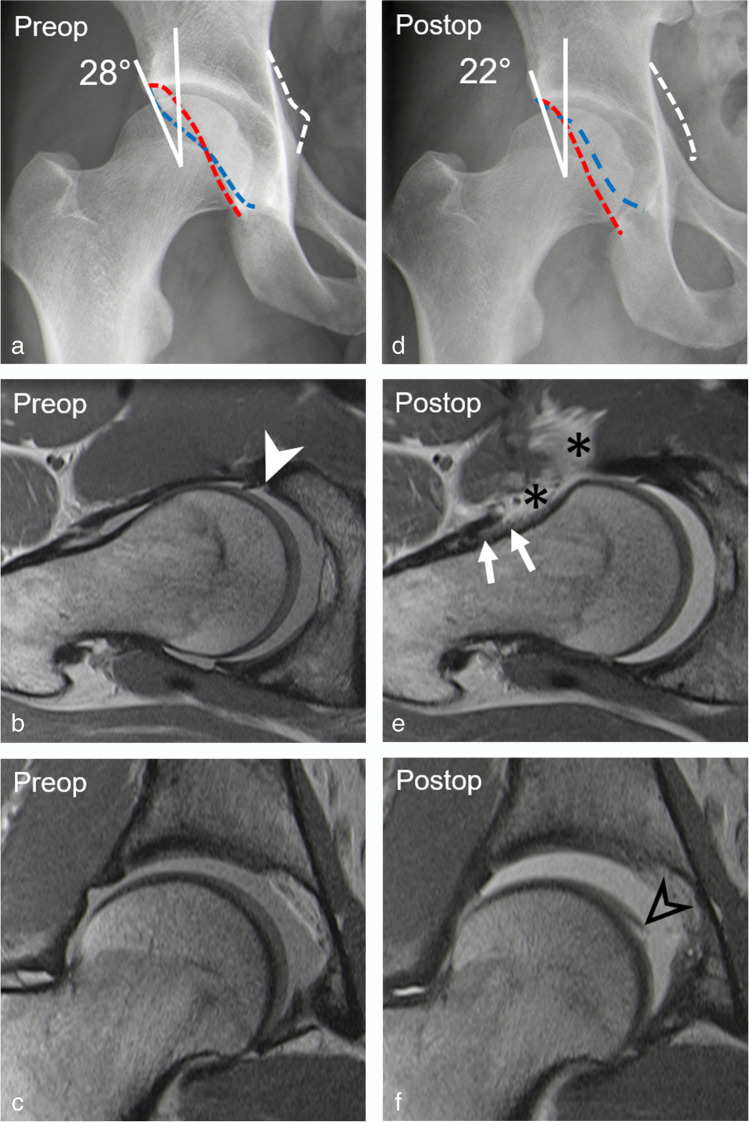
A 29-year-old patient presenting with residual pain 2 years after arthroscopic trimming of the retroverted acetabulum, labrum refixation, and cam resection. **a** Preoperative AP pelvis view shows normal lateral coverage (LCE: 28°) but a retroverted acetabulum. **b** Preoperative axial-olbique and **c** coronal PD-w TSE images (repetition time/echo time, 2460 ms/13 ms) show anterior labrum tear (filled arrowhead) and intact cartilage. **d** Postoperative AP pelvis view shows deficient acetabular coverage (LCE: 22°) following acetabular overcorrection. **e** Postoperative axial-oblique and **f** coronal PD-w TSE images (repetition time/echo time, 2460 ms/13 ms) show capsular adhesions (white arrows) and capsular defect (asterisks) and new femoral cartilage delamination (black arrowhead). Note: ischial spine sign (white dotted line)

The prevalence of hip dysplasia (LCE < 25°) increased significantly (both readers, *p* = 0.01) in reader 1/2 from 2% (1/46)/4% (2/46) preoperatively to 20% (9/46)/22% (10/46) postoperative, corresponding to a difference of 18% (95% CI, 7–29%) (Figs. 1, 2). This was reflected by a significant (both readers *p* < 0.001) decrease in mean LCE angle of 3 ± 4° for reader 1 (95% CI, 2–5°) and reader 2 (95% CI, 2–4°) (Table 2).

**Fig. 3:**
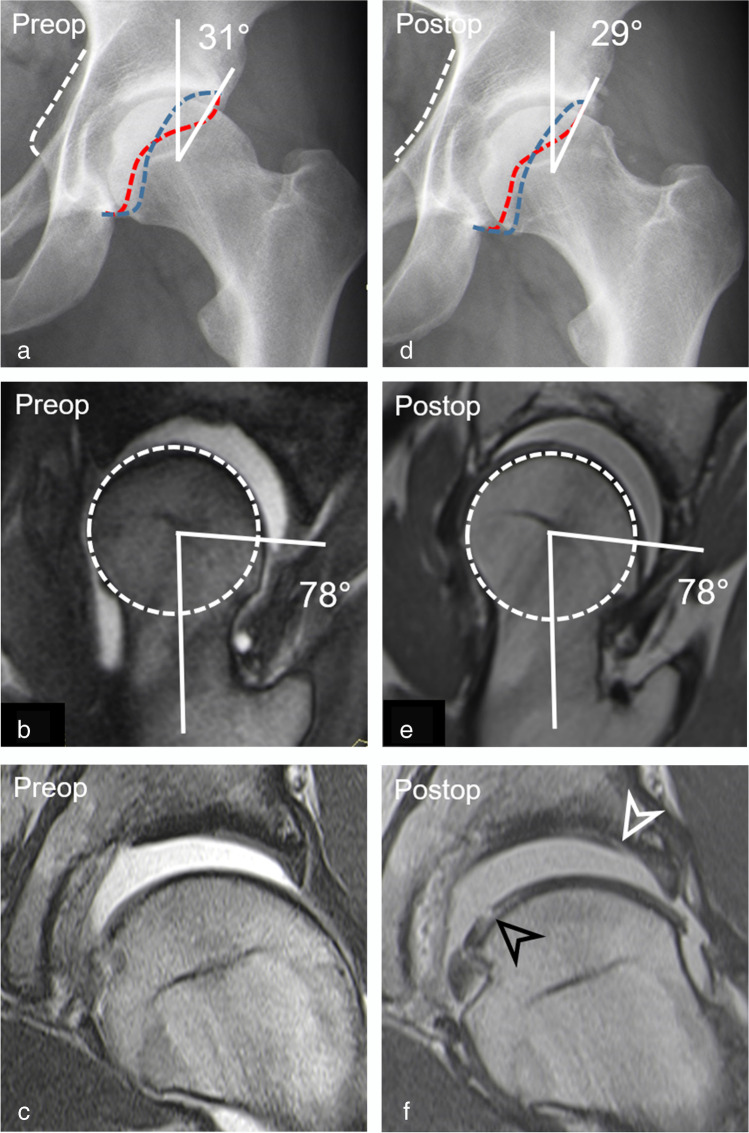
A-26 year-old man presenting with groin pain 3 years after arthroscopic acetabular rim trimming, labrum refixation, and cam resection. **a** Preoperative AP pelvis view shows acetabular retroversion with normal lateral coverage (LCE: 31°) and a cam deformity. **b** Preoperative radial reformatted image (3D T2-w true FISP, repetition time/echo time, 4.7 ms/2 ms) shows a cam deformity (alpha angle: 78°). **c** Preoperative coronal MR arthrogram (T1-w TSE sequence; 450 ms/ 12 ms) shows no chondral damage. **d** Postoperative radiograph shows less pronounced crossover sign. **e** Postoperative radial image (3D PD-w SPACE, 1100 ms/41 ms) shows incomplete cam resection with residual cam deformity postero-superiorly. **f** Postoperative coronal T1-w TSE image (450 ms/12 ms) shows progressive cartilage damage with new acetabular (white arrowhead) and femoral (black arrowhead) chondral damage. Note: Anterior acetabular wall (red dotted line), posterior acetabular wall (blue dotted line), ischial spine sign (white dotted line), femoral head (white dotted circle)

**Fig. 4:**
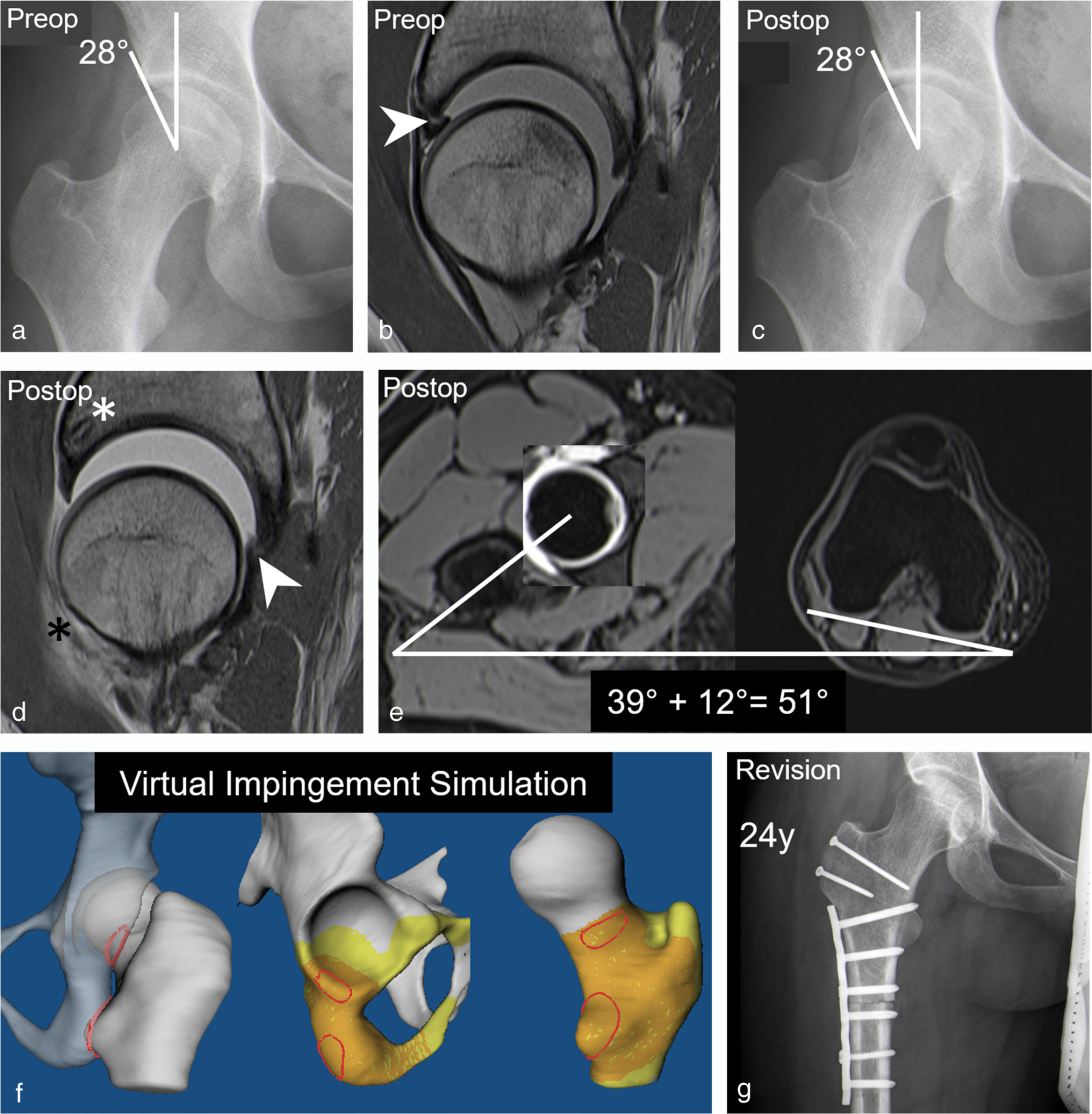
A 24-year-old patient presenting with persisting pain 1.5 years after arthroscopic labrum refixation and cam resection. **a** Preoperative AP pelvis view shows no acetabular deformity. **b** Preoperative sagittal PD-w TSE image (repetition time/echo time, 2460 ms/13 ms) shows anterior labrum tear (arrowhead). **c** Postoperative AP pelvis view shows no obvious deformity. **d** Postoperative sagittal PD-w TSE image (2460 ms/13 ms) shows intact anterior labrum after refixation with suture anchors (white asterisk) but new labrum tear posterior (arrowhead). Anterior capsule defect with adhesion (black asterisk). **e** Postoperative axial T1-w VIBE dixon sequence (6.7 ms, 2.4 and 4.8 ms) shows increased femoral torsion (51°). **f** Postoperative MRI-based 3D impingement simulation was performed which confirmed an ischiofemoral impingement conflict occurring at the lesser trochanter, the posterior acetabulum, and the ischial tuberosity (red areas) during combined extension, external rotation, and adduction. **g** Postoperative radiograph following subsequent open surgical hip dislocation and subtrochanteric derotational osteotomy for treatment of ischiofemoral impingement

Readers 1 and 2 found a significant (both readers, *p* < 0.001) decrease of 55% (95% CI, 41–69%) and 54% (95% CI, 40–68%) in the prevalence of a cam deformity from 83% (38/46) and 76% (35/46) preoperatively to 28% (13/46) and 22% (10/46) postoperatively (Fig. 3). Accordingly, a mean decrease in alpha angles of 11 ± 11° (95% CI, 7–14°; *p* < 0.001) and 10 ± 11° (95% CI, 7–14°; *p* < 0.001) was observed by readers 1 and 2, respectively (Table 2). Mean femoral torsion was 24 ± 11° for reader 1 and 26 ± 12° for reader 2. The prevalence of exccessivley high femoral torsion (> 35°) was 24% (11/46) for reader 1 and 20% (9/46) for reader 2 (Table 2, Fig. 4). The prevalence of femoral retrotorsion (< 0°) was 4% (2/46) for both readers (Table 2).

### Prevalence of intra-articular lesions

Postoperatively, 96% (reader 1, 44/46) and 98% (reader 2, 45/46) of patients had any intra-articular lesion ranging from 20% (reader 1, 9/46) and 17% (reader 2, 8/46) for femoral cartilage lesions to 65% (reader 1, 30/46) and 80% (reader 2, 37/46) for acetabular cartilage lesions. Preoperatively, readers 1 and 2 detected a labrum lesion in 93% (43/46) and 98% (45/46) of patients. Postoperatively, the prevalence decreased to 48% (22/46) and 50% (23/46) corresponding to a significant decrease of 45% (95% CI, 31–59%, *p* < 0.001) and 48% (95% CI, 34–62%; *p* < 0.001) for readers 1 and 2, respectively (Table 2).

Both readers detected progressive cartilage damage in 37% (17/46) of the patients.

The prevalence of extensive cartilage lesions > 2 h increased from 24% (11/46) and 20% (9/46) preoperatively to 37% (17/46) and 41% (19/46) for reader 1 (difference of 13%, 95% CI, 3–23%; *p* = 0.07) and reader 2 (difference of 21%, 95% CI, 9–33%; *p* = 0.01) on postoperative imaging (Table 2).

A capsular defect was found postoperatively in 48% (22/46) and 59% (27/47) of patients for readers 1 and 2, respectively. Capsular adhesions were present postoperatively in 39% (18/46) and 48% (22/46) of patients for reader 1 and reader 2, respectively (Fig. 2).

Femoral neck fractures or femoral head necrosis were not detected.

### Interrater reliability

Interrater reliability for preoperative diagnosis of osseous deformities ranged from κ of 0.66 for hip dysplasia to κ of 0.91 for acetabular retroversion. Postoperatively, κ ranged from 0.60 for presence of cam deformity to 1.00 for diagnosis of femoral retrotrosion. Interrater reliability for detection of preoperative intra-articular lesions ranged from κ of 0.51 to 0.85 for diagnosis of acetabular/femoral cartilage lesions. Postoperatively, interrater reliability ranged from 0.52 for diagnosis of acetabular cartilage lesions to 0.82 for diagnosis of capsular adhesions (Table 3).

**Table 3:** Measure of interobserver agreement

Parameter	Baseline	*p* value	Postoperative	*p* value
	ĸ value		ĸ value	
Osseous deformity				
Hip dysplasia (LCE < 25°)	0.66	< 0.001	0.80	< 0.001
Severe overcoverage (LCE > 40°)	0.80	< 0.001	0.62	< 0.001
Acetabular retroversion	0.91	< 0.001	0.66	< 0.001
Cam deformity	0.67	< 0.001	0.60	< 0.001
Increased femoral torsion (> 35°)	–	–	0.62	< 0.001
Decreased femoral torsion (< 5°)	–	–	1.00	< 0.001
Intra-articular lesion				
Postoperative labrum tear	–	–	0.78	< 0.001
Acetabular cartilage lesion	0.51	< 0.001	0.52	< 0.001
Femoral cartilage lesion	0.85	< 0.001	0.78	< 0.001
Progressive cartilage damage	–	–	0.72	< 0.001
Capsular defect	–	–	0.70	< 0.001
Capsular adhesion	–	–	0.82	< 0.001

*LCE* lateral center edge angle

### Association between osseous deformities and intraarticular lesions

**Fig. 5:**
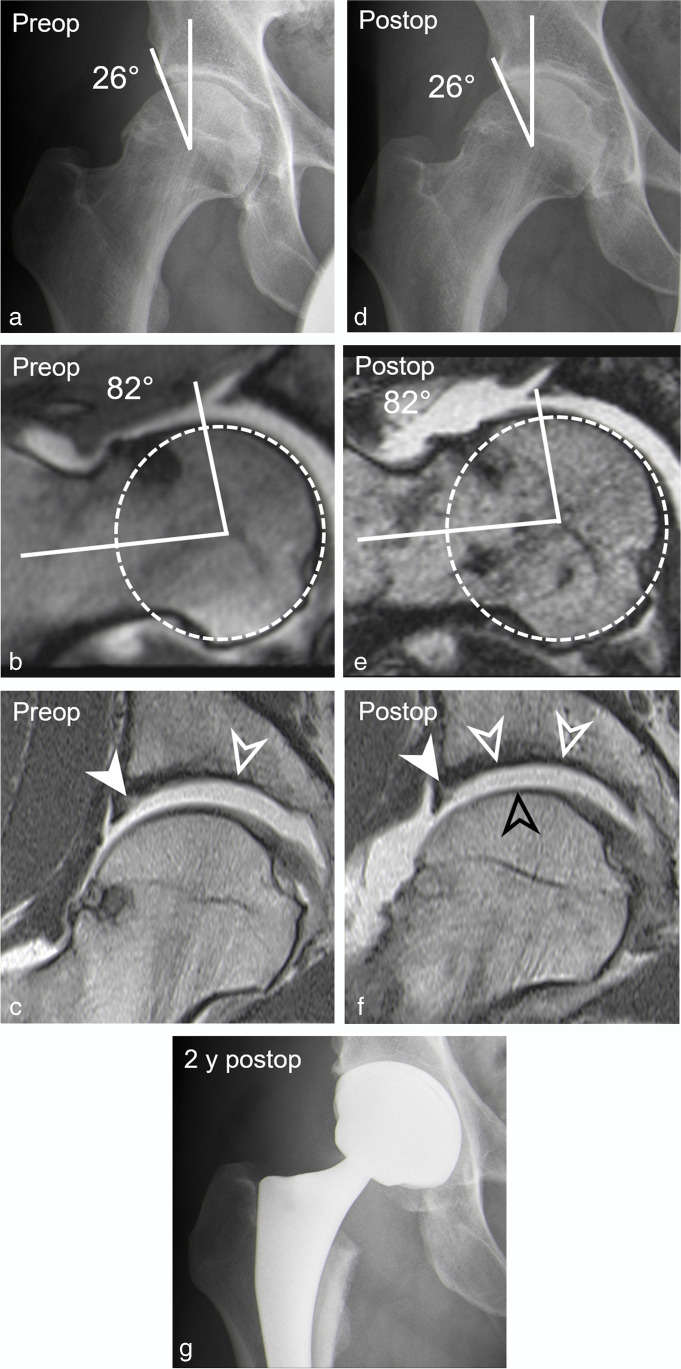
A 45-year-old patient presenting with aggravated groin pain 1 year after arthroscopic cam resection and labrum debridement. **a** Preoperative AP pelvis view shows normal lateral coverage (LCE: 26°) and a cam deformity but a preserved joint space. **b** Preoperative radial reformatted image (3D T2-w true FISP, repetition time/echo time, 4.7 ms/2 ms) shows superior cam deformity (alpha angle: 82°). **c** Preoperative coronal T1-w TSE image (450 ms/12 ms) shows extensive cartilage damage of the central acetabulum (white arrowhead) and labrum tear (filled arrowhead). **d** Postoperative AP pelvis view with normal acetabular coverage (LCE: 26°) and joint space narrowing. **e** Postoperative radial reformatted image (3D T2-w true FISP, 4.7 ms/2 ms) shows residual cam deformity due to undercorrection (alpha angle: 82°). **f** Postoperative T1-w TSE image (450 ms/ and 12 ms) shows complete loss of acetabular cartilage (white arrowheads) and new femoral cartilage thinning (black arrowhead) indicating progressive cartilage damage. Residual labrum tear (filled arrowhead). **g** Postoperative AP pelvis view following total hip replacement 8 months after postoperative MR arthrogram. Note: femoral head (white dotted circle)

**Fig. 6:**
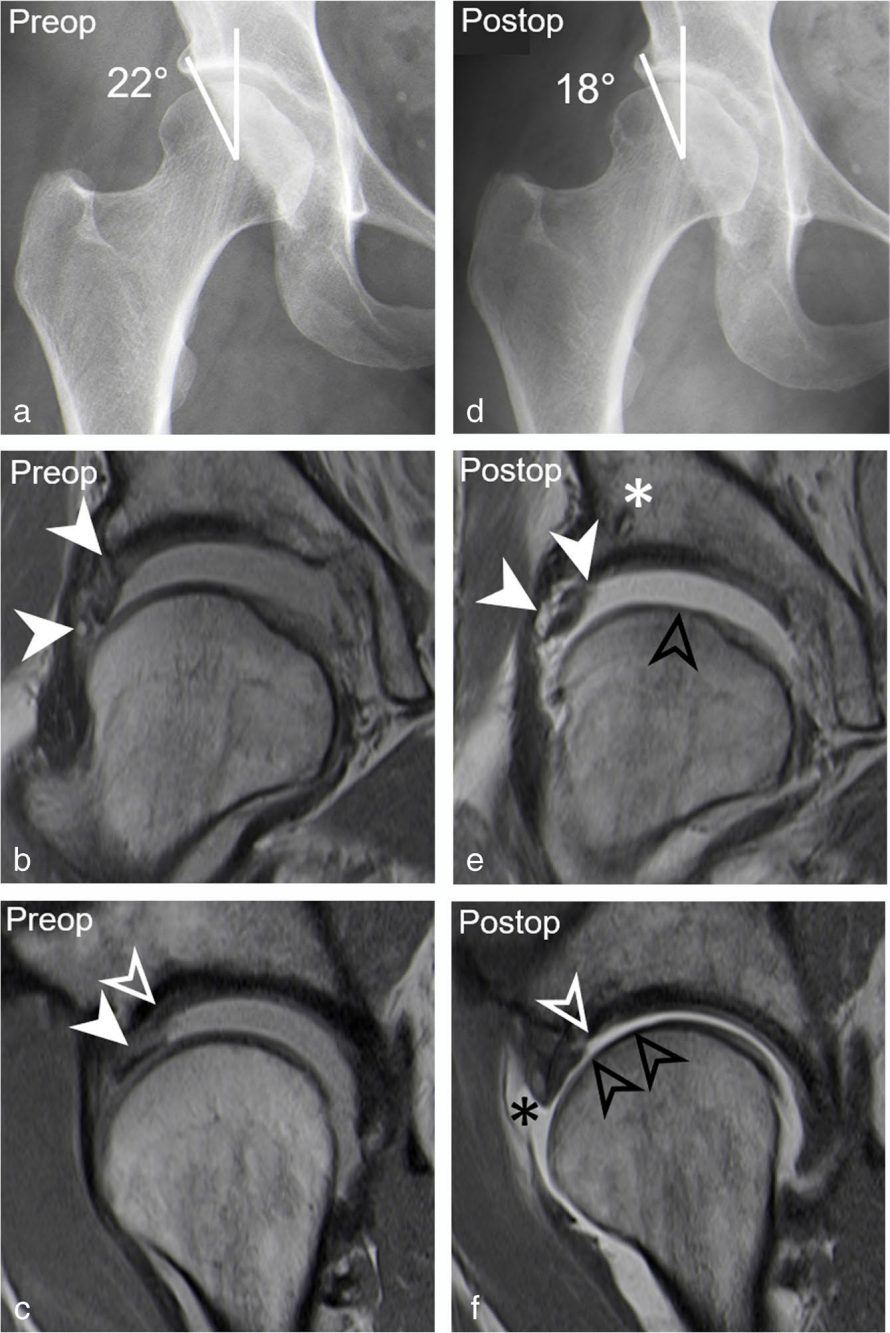

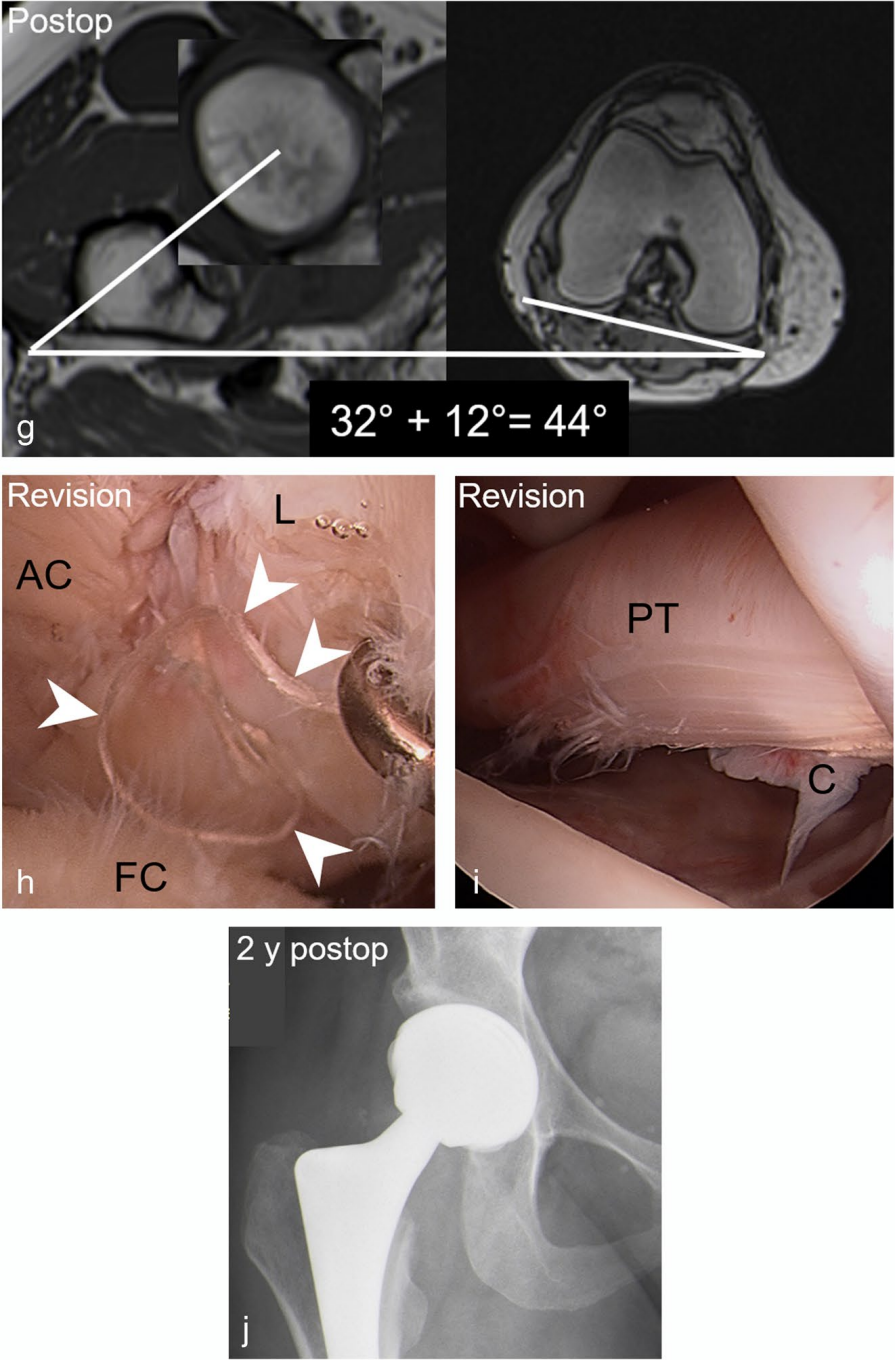
A 38-year-old woman presenting with persisting pain 1 year after arthroscopic acetabular rim trimming and labrum refixation. **a** Preoperative AP pelvis view shows dysplasia with decreased lateral coverage (LCE: 22°). **b** Preoperative coronal PD-w TSE image (repetition time/echo time, 2460/13 ms) shows labrum tear with paralabral cyst (filled arrowheads). **c** Preoperative sagittal PD-w TSE image (2460 ms/13 ms) shows extensive acetabular cartilage delamination (white arrowhead) with adjacent labrum tear (filled arrowhead). **d** Postoperative AP pelvis view shows further reduced lateral coverage (LCE: 18°) after acetabular rim trimming. **e** Postoperative coronal PD-w TSE image (2460/13 ms) shows retear of labrum (filled arrowheads) following labrum refixation with suture anchors (asterisk) and new femoral cartilage thinning (black arrowhead). **f** Postoperative sagittal PD-w TSE image (2460 ms/13 ms) shows excessive acetabular cartilage defect (white arrowhead), new femoral cartilage defect (black arrowheads), and a capsular defect (black asterisk). **g** Postoperative axial T1-w TSE image (540 ms/19 ms) and axial T1-w VIBE DIXON of the knee (6.7 ms/2.4 and 4.8 ms) show excessively high femoral torsion of 44°. **h** At revision hip arthroscopy the retear of the labrum (L) was confirmed with loose sutures (white arrowheads) and acetabular (AC) and femoral (FC) chondral damage. **i** Intraoperative remnants of the anterior joint capsule (C) can be seen along with an intraarticular course of the psoas tendon (PT). **j** The patient had persisting pain following revision hip arthroscopy and underwent subsequent total hip replacement

Multivariate analysis showed that extensive preoperative cartilage damage > 2 h (OR [odds ratio] 7.72, 95% CI 1.52–50.28; *p* = 0.02) and an incremental increase in postoperative alpha angles (OR 1.18, 95% CI 1.03–1.44; *p* = 0.04) was associated with progressive cartilage damage (Table 4, Figs. 3, 5). Postoperative hip dysplasia was the only parameter associated (OR 5.13, 95% CI 1.07–37.76; *p* = 0.04) with presence of postoperative labrum lesions (Table 5, Fig. 6).

**Table 4:** Association between demographic factors and imaging parameters with progressive cartilage damage on postoperative MR arthrograms

Parameter	Progressive cartilage damage						
	Yes	No	Difference	Odds ratio univariate	*p* value	Odds ratio multivariate	*p* value
	(*n* = 17)	(*n* = 29)					
Age	31 ± 10	28 ± 9	3 (− 3–9)	1.04 (0.97–1.11)	0.256		
Preoperative cartilage lesion > 2 h	41% (7)	14% (4)	27% (14–40%)	4.38 (1.08–20.02)	0.038	7.72 (1.52–50.28)	0.02
PreoperativeTönnis > 0	47% (8)	21% (6)	26% (13–39%)	3.41 (0.94–13.24)	0.067		
Postoperative LCE (°)	29 ± 7	32 ± 6	3 (− 1–7)	0.97 (0.87–1.07)	0.536		
Dysplasia (LCE < 25°)	35% (6)	10% (3)	25% (13–38%)	4.73 (1.05–25.77)	0.043	6.14 (0.89–58.64)	0.08
Severe overcoverage (LCE > 40°)	12% (2)	14% (4)	2% (− 2–6%)	0.83 (0.11–4.82)	0.844		
Postoperative acetabular retroversion	6% (1)	0% (0)	6% (− 1–13)	n.a	n.a		
Postoperative cam deformity	47% (8)	17% (5)	30% (17–43%)	4.27 (1.13–17.67)	0.032	0.09 (0.00–3.53)	0.20
Alpha angle (°)	66 ± 11	57 ± 10	9 (3–16)	1.09 (1.02–1.17)	0.005	1.18 (1.03–1.44)	0.04
Postoperative femoral torsion (°)	22 ± 11	25 ± 10	3 (− 4–9)	0.98 (0.92–1.03)	0.402		
Increased femoral torsion (> 35°)	18% (3)	28% (8)	10% (1–19%)	0.56 (0.11–2.33)	0.438		
Decreased femoral torsion (< 0°)	12% (2)	0% (0)	12% (3–21%)	n.a	n.a		
Postoperative capsular defect	29% (5)	59% (17)	30% (17–43%)	0.29 (0.08–1.02)	0.052		
Postoperative adhesions	41% (7)	38% (11)	3% (− 2–8%)	1.15 (0.33–3.90)	0.828		

*LCE* lateral center edge angle

**Table 5:** Association between demographic factors and imaging parameters with postoperative labrum lesions on postoperative MR arthrograms

Parameter	Postoperative labrum lesion				
	Yes (*n* = 22)	No (*n* = 24)	Difference	Odds ratio univariate	*p* value
Age	31 ± 10	27 ± 10	4 (− 3–9)	1.034 (0.97–1.10)	0.28
Preoperative cartilage lesion > 2 h	36% (8)	13% (3)	23% (11–35)	4.00 (0.97–20.76)	0.06
Preoperative Tönnis > 0	41% (9)	21% (5)	20% (8–32%)	2.63 (− 0.31–2.33)	0.14
Postoperative LCE (°)	30 ± 8	31 ± 5	1 (− 5–3)	0.99 (0.89–1.07)	0.62
Dysplasia (LCE < 25°)	32% (7)	8% (2)	24% (12–36%)	5.13 (1.07–37.76)	0.04
Severe overcoverage (LCE > 40°)	14% (3)	13% (3)	1% (− 2–4%)	1.11 (0.18–6.61)	0.91
Postoperative acetabular retroversion	5% (1)	0% (0)	5% (− 1–11%)	n.a	n.a
Postoperative cam deformity	32% (7)	25% (6)	7% (0–14%)	1.40 (− 0.96–1.66)	0.61
Alpha angle (°)	61 ± 11	60 ± 11	1 (− 7–6)	1.01 (0.95–1.06)	0.82
Postoperative femoral torsion (°)	24 ± 11	24 ± 10	0	1.00 (0.95–1.06)	0.99
Increased femoral torsion (> 35°)	18% (4)	29% (7)	11% (2–20%)	0.54 (0.12–2.12)	0.38
Decreased femoral torsion (< 0°)	5% (1)	4% (1)	1% (− 2–4%)	n.a	n.a
Postoperative capsular defect	41% (9)	54% (13)	13% (3–23%)	0.59 (0.18–1.87)	0.37
Postoperative adhesions	41% (9)	38% (9)	3% (− 2–8%)	1.15 (0.35 3.82)	0.81

*LCE* lateral center edge angle

## Discussion

Despite the fact that the number of patients with hip pain following arthroscopic FAI surgery are increasing secondary to the rise of hip arthroscopy, studies investigating osseous deformities and intra-articular lesions on postoperative imaging are sparse [10].

Most patients had osseous deformities of the acetabulum or femur on postoperative imaging (74% for reader 1 and 65% for reader 2). Prevalence of hip dysplasia increased from 2% and 4% preopervatively to 20% and 22% postoperatively for two readers (*p* = 0.01), secondary to overcorrection at the acetabular rim. Cadaveric studies have shown that excessive acetabular rim trimming can dramatically increase contact pressures with potentially detremential effects even in non-dysplastic hip joints [27]. Insufficient acetabular coverage on radiographs (LCE < 22°, hazard ratio 5.4) or excessive osseous debridement on MRI reportedly increases the long-term failure rate and impairs short-term improvement after FAI surgery [2]. Identification of these patients is important as they may require subsequent periacetabular osteotomy to correct iatrogenic acetabular undercoverage [7]. In the present study, a postoperative LCE angle < 25° was associated (OR 5.13, *p* = 0.04) with the presence of postoperative labrum lesions which supposedly result from the increased stress load. In addition, excessively high femoral torsion can aggravate the overload to the acetabulum due to anterior translation of the femoral head secondary to an ischiofemoral impingement conflict [28–30]. In the present study, the prevalence of exccessivley high femoral torsion (> 35°) measured at the level of the lesser trochanter was 24% for reader 1 and 20% for reader 2, considerably higher than the 12% reported in 538 patients with FAI and hip dysplasia using the same measurement method [31]. Although indications are still evolving, early studies report favorable outcome following derotational osteotomies for treatment of ischiofemoral impingement and instability [29]. Defects of the hip capsule represent another potentially destabilizing factor secondary to hip arthroscopy. Identification of capsular defects is important as some patients may require revision surgery for capsular repair [32]. Similar to previous reports, the prevalence of capsular defects was high with 48% and 59% for two readers [11, 32].

Restoring a spherical femoral head neck junction is critical to achieve maximum clinical short-term outcome and residual cam deformities reportedly are among the most frequent causes for revision surgery [12, 33]. Alpha angles decreased from 71 ± 9° and 70 ± 11° preoperatively to 60 ± 11° and 60 ± 10° postoperatively for readers 1 and 2 (*p* < 0.001) following cam resection. Accordingly, 28% (reader 1) and 22% (reader 2) of the patients had a residual cam deformity using the commonly recommended threshold of 60° for the alpha angle [20]. The present study underlines the importance of a complete cam resection as an increased odds for progression of cartilage damage was observed with increasing alpha angles (OR 1.18, *p* = 0.04). Previously Ross et al. reported a mean alpha angle of 68 ± 16° corresponding to a 86% prevalence of residual cam deformity when applying a threshold of 50° in 50 patients undergoing revision hip arthroscopy. The authors stressed the importance of using dynamic virtual impingement simulation in these patients to take the entire hip anatomy into account [12]. This further includes measurement of femoral torsion as reduced femoral torsion can cause hip impingement in the absence of a cam deformity and has been reported as a risk factor for worse clinical outcome following arthroscopic FAI surgery [34, 35].

Manifest radiographic osteoarthritis is an established negative predictor for success of joint preserving surgery but pre-arthritic to early degenerative radiographic changes fail in predicting severity of already present cartilage damage [20]. Previously, it has been shown that extensive cartilage damage > 2 h on the clock face is associated with an increase risk (hazard ratio 4.6) of failure following open FAI surgery [24]. In the present study, extensive cartilage damage > 2 h was associated with increased odds (OR 7.72, *p* = 0.02) for progressive cartilage damage on postoperative MR arthrograms. This underlines the potential benefit of a detailed reporting of cartilage damage in risk stratification and surgical decision-making. In the current study, inter-reader reliabilites for imaging assessessment ranged from moderate to almost perfect (κ = 0.51 to 1) which is similar to a previous study on postoperative MR arthrographic findings after FAI surgery which reported fair to almost perfect agreement between readers (κ = 0.25 to 1) [11].

Our study has several limitations. First, due to ethical reasons, we did not include an asymptomatic control group following hip arthroscopy who underwent MR arthrography of the hip. Previous studies have shown that chondro-labral damage and capsular lesions are highly prevalent on postoperative MRI in patients with good clinical outcome [10, 11]. More specifically, Kim et al. demonstrated that the prevalence of intra-articular lesions on postoperative MR arthrography including chondro-labral and capsular lesions was comparable between patients with and without pain 13 months after arthroscopic FAI correction [11]. However, comparing pre- and postoperative MRI after arthroscopic FAI correction, Foreman et al. could show that increased depth and length of the acetabuloplasty was associated with decreased short-term improvement in pain scores.[10]. Second, due to the retrospective study design, patients did not complete pre- and postoperative questionnaires. Hence, it was not possible to assess changes in patient-reported outcome scores to evaluate which imaging findings are associated with worse clinical outcome. Overall, this highlights the importance of correlating postoperative imaging findings with clinical presentation in a given patient and the need for further research investigating their association. Third, assessment of intra-articular lesions was based on traction MR arthrography. While leg traction reportedly improves visualization of cartilage layers, the subluxation of the femoral head can make identification of capsular lesions more difficult [18]. Ideally, a direct comparison between images obtained with and without traction should be performed. which was not possible in a busy clinical setup and should be assessed in future studies.

To conclude, we found a high prevalence of osseous deformities due to over- or undercorrrection of the hip and a high prevalence of associated intra-articular lesions in patients following failed hip arthroscopy. Detection of these deformities is important as they may expose the hip to ongoing stress and can potentially lead to progressive cartilage damage and labrum lesions.

## Supplementary Information

Below is the link to the electronic supplementary material.

Supplementary file1 (DOCX 18 KB)

## Abbreviations

AP: Anteroposterior
CI: Confidence interval
FAI: Femoroacetabular impingement
LCE: Lateral center edge angle
OR: Odds ratio

## Declarations

### Guarantor

The scientific guarantor of this publication is Florian Schmaranzer, MD PhD.

### Statistics and biometry

One of the authors has significant statistical expertise.

### Methodology

• retrospective

• observational study

• performed at one institution
