# Supplementary material for: High prevalence of hip lesions secondary to arthroscopic over- or undercorrection of femoroacetabular impingement in patients with postoperative pain
Source: Eur Radiol. 2021 Nov 29;32(5):3097–111. doi: 10.1007/s00330-021-08398-4 (PMC9038890; doi:10.1007/s00330-021-08398-4)
Supplement: Supplementary file 1 — Supplementary file1 (DOCX 18 KB) [file 330_2021_8398_MOESM1_ESM.docx]

| **ELECTRONIC SUPPLEMENTARY MATERIAL**     **Supplemental Table 1. MR Sequence protocol.** | | | | | | | | | | |
| --- | --- | --- | --- | --- | --- | --- | --- | --- | --- | --- |
| Sequence | Repetition  Time (ms) | Echo  Time (ms) | Matrix | FOV (mm) | Flip  angle | Slice  Thickness (mm) | Bandwidth  (Hz/Px) | Pre-/  Postoperative | Image  orientation | Traction |
| T1-w turbo spin echo | 450 | 12 | 448 x 224 | 180 | 90 | 3 | 130 | preoperative | coronal | yes |
| T1-w FLASH | 475 | 9.8 | 448 x 224 | 180 | 60 | 3 | 70 | preoperative | coronal/ sagittal/ axial-oblique | yes |
| 3D true FISP | 4.66 | 2 | 256 x 256 x 256 | 200 | 70 | 0.8 | 501 | pre-/ postoperative | axial-oblique | yes |
| PD-w turbo spin echo | 2460 | 13 | 512 x 512 | 180 | 150 | 3 | 130 | pre-/ postoperative | coronal/ sagittal/  axial-oblique | yes |
| 3D PD-w SPACE | 1100 | 41 | 256 x 256 | 256 | variable | 1 | 501 | postoperative | axial-oblique | yes |
| T1-w turbo spin echo | 540 | 19 | 320 x 320 | 380 | 90 | 4 | 130 | postoperative | axial pelvis | no |
| T1-w VIBE DIXON | 6.7 | 2.4/ 4.8 | 320 x 320 | 380 | 10 | 3 | 470 | postoperative | axial pelvis and knee | no |
| Preoperative protocol: Multiplanar T1-w FLASH or PD-w turbo-spin echo, coronal T1-w turbo spin echo and axial-oblique 3D true FISP  Postoperative protocol: Multiplanar PD turbo-spin echo, axial-oblique 3D true FISP or 3D PD-w SPACE, axial T1-w turbo spin echo and T1-w VIBE DIXON  Abbreviation: FLASH = fast low-angle shot obtained with fat-saturation, true FISP = true fast imaging with steady-state progression, SPACE = sampling perfection with application-optimized contrasts using different flip angle evolution, VIBE = volume interpolated breath-hold-examination | | | | | | | | | | |
